# Different Dimensions of Grit as the Predictor of Job-Search Intensity and Clarity

**DOI:** 10.3389/fpsyg.2022.825509

**Published:** 2022-06-27

**Authors:** Xuan Yu, Yue Yuan, Xuhong Liu, Bin He

**Affiliations:** ^1^School of Economics and Management, Southwest Petroleum University, Chengdu, China; ^2^School of State Governance, Southwest University, Chongqing, China; ^3^Department of Police Management, Sichuan Police College, Luzhou, China

**Keywords:** grit, job-search intensity, job search clarity, perseverance of effort, consistency of interest

## Abstract

Job-search is considered as a developmental task for college students to move from campus to workplace. Based on the self-determination theory, 859 Chinese college students were selected as the study sample and hierarchical regression analysis was used to explore the perseverance of effort and consistency of interest on job-search intensity and clarity. The survey showed that the perseverance of effort has a significant positive effect on the job-search intensity, while it has no significant positive effect on job-search clarity. Consistency of interest has a significant negative effect on job-search intensity and a significant positive effect on job-search clarity. Theoretical and practical implications are discussed and the directions for future research are outlined in this study.

## Introduction

According to the Chinese Ministry of Education, more than 10 million college students have graduated in the country in 2022. The employment situation is becoming increasingly serious for college graduates. Coupled with the impact of coronavirus disease 2019 (COVID-19), a large number of industries have been hit, leading to frequent layoffs and unemployment situations in China, which has increased the employment pressure on college students (Hensher, 2020; Kawohl and Nordt, 2020). All these factors have worsened the employment status of university students, and the problem has drawn increasing attention. Job-search is considered as a developmental task for college students to move from campus to the workplace, and job-search behavior shows the level of engagement in their transition process (Dietrich et al., 2012; Kim and Lee, 2021). As new entrants to the labor market, they may have limited familiarity with the opportunities available to them (Turban et al., 2009). Many are at the early stages of forming career goals, particularly those who lack the clarity of a predefined career trajectory or strong socialization influences linked to their degree subject (Powers and Myers, 2017; Rummel et al., 2019). Students, for the first time, face these complex and difficult job-search environments, emerging with a strong fear of difficulties and some withdrawal behavior. Many of them feel confused about their career planning, career selection, and career objectives. A successful job-search requires a clear understanding of what we want to do and the degree of job-search effort (Kanfer et al., 2001; van Hooft et al., 2021). Lack of job-search intensity and clarity can influence the effectiveness of job-search behavior. Lower job-search clarity job seekers cannot effectively send job-search signals to potentially suitable employers (Côté et al., 2006). In these circumstances, an effective job-search involving important clarity and intensity, including goal-directed effort and appropriate information gathering, is argued to be crucial for securing meaningful employment (Kanfer et al., 2001; Wanberg et al., 2020; van Hooft et al., 2021).

Several studies have already revealed that job-search intensity and clarity were associated with the Big Five personality (Caldwell and Burger, 1998; Wanberg et al., 2000; Kanfer et al., 2001), proactive personality (Zacher, 2013), self-esteem (Ellis and Taylor, 1983), and self-efficacy (Saks and Ashforth, 1999; Zacher and Bock, 2014). An important aspect in personality theory should include an important aspect in addition to cognitive factors, i.e., non-cognitive factors. The important role of these non-cognitive factors (e.g., grit) is gradually being emphasized but has not been fully studied (Wong et al., 2018). In this study, we focused on “grit”, a non-cognitive factor related to motivation. It is the psychological drive that encourages hard-working, persevering, and passionate people to succeed. The concept of grit stems from the belief that enduring passion and perseverance are more important than talent. In positive psychology, grit is defined as “trait-level perseverance and passion for long-term goals” (Duckworth et al., 2007, p. 1087). In recent years, grit has received much academic attention which can positively influence goal effort and achievement and social performance, and can lead to positive outcomes (Hill et al., 2016; Bonfiglio, 2017; Li et al., 2018). Unlike other cognitive factors, grit is developed through mindset, skills, and passion (Duckworth, 2016), tends to be more aware of future career directions, and also spends more time persistently searching for jobs (Kaufman and Duckworth, 2017). It has been found that job-search is influenced by personal motivation (Kanfer et al., 2001). Studies by Deci and Ryan (1985) and Deci and Ryan (2008) concluded that there is a positive relationship between intrinsic motivation, learning, and achievement. Also, intrinsically motivated students will persist despite the difficult tasks or conditions. Self-determination theory (SDT) explains the diversity of learning strategies and academic achievement. According to SDT, individuals with higher levels of grit are more persistent in pursuing goals than their less grit peers despite setbacks, disruptions, or other forms of interference (Duckworth and Quinn, 2009; Maddi et al., 2012; Strayhorn, 2014). Thus, individuals with high grit characteristics are highly able to enhance their job-search intensity and clarity. Individuals with high grit then tend to demonstrate their determination to achieve their goals, have strong resilience in the face of difficulties, and are focused (Clark and Malecki, 2019). So, when college students are faced with a job-search, they may make a stronger determination and greater effort.

Surprisingly, despite the importance of these aspects to students’ job-search, few studies have linked them with job-search intensity and clarity. Grit comprises two dimensions, namely, perseverance of effort and consistency of interest. Given the explicit distinction in these two dimensions, it is presently indistinct whether these aspects of grit predict essential job-search intensity and clarity and whether they play the same roles in predicting these outcomes. With this in mind, we decided to use grit as a key variable and empirically test its effects on college students’ job-search intensity and clarity.

### Theoretical Background and Hypothesis

Personal motivation comes from both internal (intrinsic) and external (extrinsic). For a college student, intrinsic motivation comes from the students themselves, such as health, intelligence and talent, interest, internal motivation, and how to learn (learning style). While extrinsic motivation originates from the school environment, society, and the family (Chue and Nie, 2012). Self-determination theory measures an individual’s commitment and motivation to achieve the goals, primarily intrinsic or extrinsic. Deci and Ryan (1985, 2008) concluded that there is a positive relationship between intrinsic motivation, learning, and achievement. However, intrinsically motivated students will persist despite their difficult tasks or conditions. Grit contains enthusiasm and interest in the target or activity, and therefore may lead to higher intrinsic motivation, and previous research has also shown that grit is statically correlated with intrinsic motivation (Ryu and Yang, 2017; Han and Park, 2018).

Grit is composed of two different dimensions, i.e., indomitable effort to achieve long-term goals (perseverance of effort) and persistent passion (consistency of interest) (Duckworth and Quinn, 2009). We argue that the two dimensions of grit will affect the intensity of work search among college students in different ways (Mooradian et al., 2016; Van Doren et al., 2019). Perseverance of effort refers to the extent to which people are consistently focused on achieving their long-term aspirations. Even in the face of a complex and difficult employment environment, people with this motivation do not easily interrupt their efforts. Perseverance in job information gathering and various job-search activities (e.g., communicating with others, searching online, and submitting applications) is emphasized. Persistence is the key to achievement, much like winning a marathon under endurance (Duckworth et al., 2007). In the process, even though it is filled with boredom and disappointment, many people begin to change their life trajectory to cut their losses, while the resilient will continue to persevere. Perseverance of effort refers to an individual’s tendency to persist and keep trying when life gets difficult or frustrating. Sometimes, we may not be interested in things or enjoy the process of achieving a goal, but we can still work toward it and eventually achieve it. According to self-determination theory, students’ motivation determines their behavior. Therefore, when students are faced with employment for the first time, students with this motivational tendency can quickly adapt to the complex employment environment, patiently gather, and filter information, and influence their job-search behavior. In a blind job-search process (e.g., future career choice), the consistency of interest is emphasized. Consistency of interest refers to the degree to which a person can persist while maintaining interest and passion to achieve long-term aspirations. It emphasizes the extent to which an individual pursues a defined goal to do so. For college students who are in the job-search phase, after establishing long-term goals during the career search process, individuals have been moving in their specific direction and maintaining passion in the process. This can better help employees achieve the behaviors and plan to achieve their long-term goals and play an important role in students’ job-search.

### Perseverance of Effort and Job-Research Intensity

Job-search intensity refers to the effort and time that people devote to job-search activities (Kanfer et al., 2001). We believe that college graduates with higher perseverance of effort will bring higher job-search intensity. Accordingly, persons with a high level of perseverance of effort should be more motivated and should work harder, and they cope with setbacks and thus spend more time and energy on this process (Credé et al., 2017). For example, showing increased perseverance of effort may enable them to cope with the challenges (e.g., complex social work environment) and even setbacks associated with spending a lot of time without finding a job. They will not stop halfway, instead, they will spend more time and effort finding job-search information when they encounter difficulties, thus making their job-search activities more intense. Thus, we proposed the following hypothesis:

*Hypothesis 1:* Perseverance of effort is positively associated with job-search intensity.

### Perseverance of Effort and Job-Research Clarity

Job-search clarity is defined as a clear goal or a clear concept of the content or form of work they want to do (Wanberg et al., 2002; Côté et al., 2006). It has a positive predictive effect on job-search results. College students are in the exploratory stage of career development, where the main task is to understand themselves and their career, and initially try to determine their career development direction and goals. As an inner guide power, perseverance of effort influences individual actions. They think that effort can be one of the effective approaches to improving ability, intelligence, and experiences (Zeng et al., 2016). Individuals would be persistent on the tasks even when they encounter challenges and difficulties. So, when individuals search for a new job, they will try to search and discover the kind of work and find ways to work, eventually with better clarity on the job-search. Therefore, we proposed the following hypothesis:

*Hypothesis 2:* Perseverance of effort is positively associated with job-search clarity.

### Consistency of Interest and Job-Search Intensity

Consistency of interest refers to the trait of always remaining enthusiastic and interested in goals for a long period closely related to career performance of positive outcomes (Robertson-Kraft and Duckworth, 2014; for a meta-analysis, see Credé et al., 2017). It is reflected in the consistency of the long-term goals of the job-search process. Clarity and enthusiasm for long-term goals require college students to study harder and search for the path needed to reach their goals and spend time on searching and planning, which is consistent with job-search intensity. Therefore, we proposed the following hypothesis:

*Hypothesis 3:* Consistency of interest is positively associated with job-search intensity.

### Consistency of Interest and Job-Search Clarity

Personality factors are one of the important factors affecting individual job-search clarity (Major et al., 2006). The consistency of interest indicates that an individual is able to have a clear understanding of the future direction when finding a job, and can maintain a long-term enthusiasm for finding a job. That is, individuals with higher interest consistency are easier to maintain a relatively positive attitude because of ignoring the job-searching environment. Positive and negative affectivity may thus influence cognitive processes involved in achieving clarity in the job-search (Salisu et al., 2020). Thus, consistency of interest can influence cognitive processes involved in achieving clarity in the job-search. Then, consistency of interest is likely to have an impact on job-search clarity as it may provide a target direction for the process. In the job-searching process, job seekers will face a lot of different job opportunities. The choices in these different jobs can get one lost, and sometimes overwhelming and may lead to career indecision (Wanberg and Muchinsky, 1992). At this point, some of the work associated with individual competence is more selected by individuals, and the consistency of interest will help to provide the target direction for compatible job opportunities (Werbel, 2000). Particularly, college graduates are eager to pursue jobs that they match and are interested in, and have a firm view of their future. The higher the consistency of interest, the individual can adhere to their consistent view, have a certain understanding of the future work direction, make job-seekers have clear goals, career, work, job type, clear ideas, and clear career goals and plan degree, and increase the job-search clarity of college students. Therefore, we proposed the following hypothesis:

*Hypothesis 4:* Consistency of interest is positively associated with job-search clarity.

**FIGURE 1:**
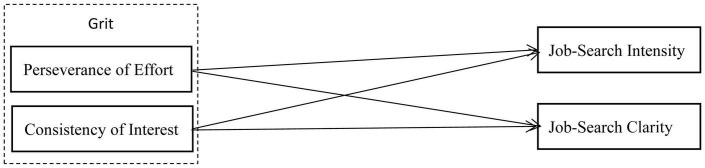
The proposed model of the study.

The conceptual model of the study is presented in Figure 1.

## Materials and Methods

### Participants and Procedures

Using the method of cluster random sampling, 1,000 students were randomly selected from the junior and senior students of a university in Chongqing, China. Our participants were recruited mainly through some remuneration and aided by explanations of information related to the participants for their fill-in answers, to help participating students understand their current situation of perseverance and to provide targeted help. To reduce common method biases, the above variables were measured in turn in 2 weeks. At the first time point (T1), data about personal information were collected, and the grit was measured in turn. A week later (T2), job-search intensity and clarity were measured. Besides, this research has set up one item separately, the last four digits of the mobile phone number, so that the data corresponding to the above variables can be effectively matched, and SPSS 21.0 and AMOS 17.0 are used to analyze and process the data.

A total of 1,000 complete questionnaires were obtained by matching the last four digits of the mobile phone number; after incomplete questionnaires have been excluded, there were 859 (85.9%) valid responses. The characteristics of the sample data are as follows: in terms of gender, 20.3% were men and 79.7% were women; in terms of GPA in last semester, “greater than 0 is less than or equal to 2”accounted for 7.7%,“greater than 2.0 is less than or equal to 2.5”accounted for 13.2%,“greater than 2.5 is less than or equal to 3.0”accounted for 18.9%,“greater than 3.0 is less than or equal to 3.5”accounted for 32.4%,“greater than 3.5 is less than or equal to 4.0”accounted for 19.8%,“greater than 4.0 is less than or equal to 4.5”accounted for 6.4%, and “greater than 4.5 is less than or equal to 5.0”accounted for 1.7%.

### Measures

In this study, we adopted the mature Western scales to measure the variables. For ensuring the consistency and applicability of the English scale in the Chinese context, the author conducted a translation-back translation procedure (Brislin, 1986). Before the formal investigation, a preliminary test was conducted on college students, and the items were modified according to their feedback.

#### Grit

Grit was measured with an 8-item scale developed by Duckworth and Quinn, 2009, and includes two dimensions, namely, consistency of interest (e.g., “I often set a goal but later choose to pursue a different one”) and perseverance of effort (e.g., “I finish whatever I begin”). Participants indicated the extent to which they agreed or disagreed on each item (1 = not like me at all; 5 = very much like me). Cronbach’s alpha for consistency of interest subscale and perseverance of effort were 0.823 and 0.834, respectively.

#### Job-Search Intensity

Job-search intensity was measured using a 10-item scale developed by Blau (1993). Participants indicated the frequency with which they have done 10 different degrees of job-search activities over the past 2 weeks (1 = Never, 5 = Very often). A sample item was, “Talked to previous employers or people I used to work with about possible job leads”. The Cronbach’s alpha for this scale was 0.776.

#### Job-Search Clarity

Job-search clarity was measured using a 4-item scale developed by Stumpf et al. (1983). Participants indicated the extent to which they agreed or disagreed on each item (1 = strongly disagree; 5 = strongly agree). A sample item was, “I need help deciding if I should make a career change”. The Cronbach’s alpha for this scale was 0.755.

#### Control Variables

Based on previous studies, we collected the general demographic information about the participants, including gender, age, and GPA in the last semester.

## Results

### Descriptive Statistics

Table 1 presents the descriptive statistics and correlations for the study variables. Perseverance of effort correlated moderately with job-search intensity (*r* = 0.136, *p* < 0.01), and is not correlated with job-search clarity (*r* = − 0.001, *p* > 0.01). Consistency of interest correlated slightly with job-search intensity (*r* = − 0.080, *p* < 0.05) and job-search clarity (*r* = 0.084, *p* < 0.05). Besides, job-search clarity correlated moderately with job-search intensity (*r* = 0.231, *p* < 0.01). Gender correlated slightly with job-search clarity (*r* = 0.073, *p* < 0.05).

**TABLE 1:** Descriptive statistics and correlations of study variables.

Variables	*M*	*SD*	1	2	3	4	5	6
1.Gender	1.800	0.402	1					
2.Age	2.590	0.932	0.051	1				
3.GPA in last semester	3.700	1.388	0.073*	0.031	1			
4.Consistency of interest	3.137	0.6948	0.000	0.029	–0.025	1		
5.Perseverance of effort	3.482	0.628	–0.046	–0.006	0.042	−0.073*	1	
6.Job-search clarity	0.888	0.256	0.073*	0.042	–0.035	0.084*	–0.001	1
7.Job-search intensity	0.626	0.309	–0.043	0.034	0.012	−0.080*	0.136**	0.231**

N = 859, *p < 0.05, **p < 0.01.

### Regression Analysis

Tables 2 and 3 present the findings of the main effect test. Perseverance of effort has the significant positive effect on job-search intensity (*B* = 0.063, SE = 0.017, *t* = 3.791, *p* < 0.001) and no significant positive effect on job-search clarity (*B* = 0.004,SE = 0.014, *t* = 0.293, *p* > 0.05), which supports hypothesis 1, but does not support hypothesis 2. Consistency of interest has the significant negative effect on job-search intensity (*B* = − 0.032, SE = 0.015, *t* = − 2.120, *p* < 0.05) and significant positive effect on job-search clarity (*B* = 0.030, SE = 0.013, *t* = 2.426, *p* < 0.05), which supports hypothesis 4, but does not support hypothesis 3.

**TABLE 2:** Results of regression analysis toward job-search intensity.

Model		Unstandardized coefficient		Standardized coefficient	T	p	Collinear statistics	
		B	Standard error	Beta			Tolerance	VIF
1	(Constant)	0.646	0.060		10.719	0.000		
	Gender	–0.035	0.026	–0.046	–1.342	0.180	0.992	1.008
	Age	0.012	0.011	0.036	1.061	0.289	0.997	1.003
	GPA in last semester	0.003	0.008	0.015	0.426	0.671	0.994	1.006
2	(Constant)	0.522	0.098		5.297	0.000		
	Gender	–0.030	0.026	–0.040	–1.169	0.243	0.990	1.010
	Age	0.013	0.011	0.039	1.152	0.250	0.996	1.004
	GPA in last semester	0.002	0.008	0.007	0.202	0.840	0.991	1.009
	Consistency of interest	−0.032*	0.015	–0.072	–2.120	0.034	0.993	1.007
	Perseverance of effort	0.063***	0.017	0.129	3.791	0.000	0.991	1.009

N = 859, *p < 0.05, ***p < 0.001. Model 1 represents the regression analysis between the control variables and the job-search intensity, and Model 2 indicates the main effects regression analysis after adding the independent variables (consistency of interest and perseverance of effort).

**TABLE 3:** Results of regression analysis toward job-search clarity.

Model		Unstandardized coefficient		Standardized coefficient	T	p	Collinear statistics	
		B	Standard error	Beta			Tolerance	VIF
1	(Constant)	0.803	0.050		16.110	0.000		
	Gender	0.047*	0.022	0.074	2.178	0.030	0.992	1.008
	Age	0.011	0.009	0.040	1.163	0.245	0.997	1.003
	GPA in last semester	–0.008	0.006	–0.041	–1.214	0.225	0.994	1.006
2	(Constant)	0.693	0.082		8.446	0.000		
	Gender	0.048*	0.022	0.075	2.195	0.028	0.990	1.010
	Age	0.010	0.009	0.037	1.095	0.274	0.996	1.004
	GPA in last semester	–0.007	0.006	–0.040	–1.165	0.244	0.991	1.009
	Consistency of interest	0.030*	0.013	0.083	2.426	0.015	0.993	1.007
	Perseverance of effort	0.004	0.014	0.010	0.293	0.770	0.991	1.009

N = 859, *p < 0.05. Model 1 represents the regression analysis between the control variables and the job-search clarity, and Model 2 indicates the main effects regression analysis after adding the independent variables (consistency of interest and perseverance of effort).

## Discussion

The school-to-work transition has become increasingly severe and complex, suggesting the need for a high-quality, self-regulatory approach to job-search. To better understand the school-to-work transition, it is critical to integrate job-search and intrapersonal motivation. While the relationship between personality traits and job-search intensity and clarity has been extensively documented in the literature (Côté et al., 2006; Bao and Luo, 2015), the potential impact of grit as a new personal motivational disposition has been overlooked. Meanwhile, there has been controversy about whether grit is a first-order or a higher-order construct composed of two lower orders (Salisu et al., 2020). For instance, Mosanya (2021) argued that grit is a complete, independent construct that cannot be divided into two dimensions, but in contrast, some studies have argued that perseverance can include two different dimensions (Salisu et al., 2020). In the present study, we believe that grit can be explored by dividing it into two dimensions. We found that people with high consistency of interest (rather than the perseverance of effort) exhibited higher job-search clarity, whereas people with high effortful perseverance (rather than the consistency of interest) exhibited higher job-search intensity. Taken together, previous studies and our findings emphasize the utility of distinguishing grit into two components (consistency of interest and perseverance of effort) rather than conceptualizing grit as a whole. Then, this study demonstrates that the perseverance of effort and consistency of interest are the antecedents of job-search intensity and clarity. Theorists have suggested that non-cognitive personality factors play an important role when attempting to link personality to specific interest criteria, and grit is considered to be an example of such a variable. Job-search intensity and clarity are important factors in the individual job-search process, and it is important to examine their influences. In this study, perseverance of effort and consistency of interest as a personality proved to be useful factors on the dispositional basis of job-search intensity and clarity.

Our findings provide new insights into the relationship between grit and job-search intensity and clarity. Specifically, grit can be regarded as two different factors, perseverance of effort and consistency of interest, and the two factors of grit are found to have different relationships on outcomes. The purpose of this study was to investigate the effects of grit (perseverance of effort and consistency of interest) on job-search intensity and clarity. Among the control variables, we found no effect of age on our results, which may be because we mainly targeted students around 20-22 years old, juniors, and seniors. While gender affects job-search clarity, after controlling the variable gender, we found that (1) there is a positive relationship between perseverance of effort and job-search intensity, while the relationship between perseverance of effort and job-search clarity is not confirmed. (2) There is a negative relationship between consistency of interest and job-search intensity, and a positive correlation and causal link between consistency of interest and job-search clarity.

First, participants with higher perseverance of effort reported higher job-search intensity, supporting hypothesis 1. This result suggests that grittier students have been shown to spend more time on studying (Cross, 2013). They are more likely to devote energy and time to their studies and job-search, which in turn can have a significant impact on their career success. Whether faced with the depth and difficulty of a complex work environment or the need for significant expertise, students have the “perseverance of effort” to achieve their desired job-search outcomes through relentless effort. Our data also suggest that perseverance of effort is not significantly associated with job-search clarity. This result differs from our hypothesis and may be explained by the fact that job-search clarity emphasizes the clarity of students’ career plans, choices, and goals, while students with strong “perseverance of effort” motivation focus more on their efforts. They do not give up on any possible opportunity, search extensively in the job market, and even try once for every job. Perseverance is not significantly related to goal setting.

In terms of another dimension of grit, consistency of interest is a predictor of higher job-searching clarity and lower job-searching intensity among students. Students with consistency of interest have maintained their initial enthusiasm for their goals throughout the job-search process. Compared with students who do not have this personality, they do not change their goals because they encounter difficulties in the job-searching process. They will remain interested in the goals until they complete the established job-search and eventually achieve a satisfactory future job-searching direction. The conclusion that higher consistency of interest brings about higher job search clarity is the same as our hypothesis 4. Students with high consistency of interest understand their intrinsic motivation for job-search, have clear interest, and can filter the types and careers they want to pursue in the job-search process, leading to more goal clarity. Interestingly, contrary to our hypothesis, the findings revealed that consistency of interest reduced our job-search intensity. The reason for the result may be that students with a high-interest inconsistent job-search can have a certain job target when they are looking for a job, so they focus less on other job-search directions and more on their target directions during the job-search, so this reduction in blind search brings less job-search content for students. So consistency of interest is negatively related to job-search intensity.

People who hold the theory of non-exhaustion of willpower believe that they can continue to do more work even after working long hours (Job et al., 2010). This has a conceptual overlap with an established psychological construct, grit. Grit is conceptualized as a relatively stable trait that is considered to be malleable. It may remain stable if left unchallenged or unreflected, but it can also be changed through interventions (Sun et al., 2019). Our study found that students with higher perseverance of effort were able to collect and find more job-related information, focus more on job-search intensity, and ignore job-search clarity compared to students with the consistency of interest. Compared to students with higher perseverance of effort, employees with higher consistency of interest can quickly identify their job-search direction and goals, increasing job-search clarity while reducing job-search intensity and reducing student stress. However, we also note that both perseverance of effort and consistency of interest are important indicators that affect students’ job-search process, and by categorizing and integrating the cultivation of these two types of students, we can effectively help students allocate and utilize their strengths reasonably when facing problems such as job-search difficulties. The findings have implications for various stakeholders involved in university-to-work transitions, most directly students/graduates, career services at universities, and employers. Career actors, such as the students and graduates in our study, take individual responsibility for gathering career-relevant information through the goal-directed job-search. In contrast to previous research, the present study recommends intrinsic motivation as main influencing factor. Our findings show that the two different dimensions of grit work in different ways. For university careers advisers, the findings imply adopting different means for effective intervention, encouraging university students to pay attention to their inner motivation, pay attention to their interest and perseverance, and make targeted changes for future job hunting, either to increase the availability and visibility of opportunities or to encourage individual career exploration activities. In particular, the consistency of interest plays an important role in the job-searching process. As the interests and goals of college students are not clear, school can provide students with related work lectures, career direction planning, internship arrangements, and other effective means, to help students build a clear understanding of social work, and eventually help them clarify their future career direction. Secondly, the high-level perseverance of efforts indicates that students can collect more relevant career information and can be fully prepared for job-search. At this stage, we can help students improve by cultivating their ability to collect and screen materials. According to our findings, the honing of these two dimensions will lead to a more successful college-to-work transition into the career stage. At the same time, we also need to pay attention to an important control variable in our study, gender. As there are differences in the clarity of students’ job-search due to gender, indicating that students with different gender characteristics have some differences in their job information collection, schools or related subjects need to take note of this phenomenon and provide training and assistance to categorize the student population.

## Limitations and Future Research

First, only self-report measures were applied, and we did not measure actual career exploration behaviors. The Grit scale is relatively transparent and, therefore, particularly vulnerable to social desirability bias. Although confidentiality was assured, some participants may have been more motivated than others by the desire to look good. Thus, this approach induces a common method bias that might inflate the observed relationships among the constructs. Psychological experiments and case studies should also be included in future research.

Second, the participants of our study are liberal arts students, with an unbalanced ratio of men and women, and the main problems they face when looking for jobs are different from those of science students. Although perseverance may also have important research implications, it cannot be explained in more depth in our article, and more attention should be paid to the generalizability of the sample in future studies.

Third, this research only explores what affects students’ job-search intensity and clarity from the individual’s perspective, while ignoring the influence of contextual factors. According to social cognitive career theory (Lent and Brown, 1996), contextual factors are important factors that affect the individual’s job-searching process. Some studies have also confirmed that parents’ behaviors, counselors’ functioning in the counseling context, etc. will affect students’ career exploration behaviors (Jiang et al., 2019), and then affect their career outcomes. Therefore, more antecedent mechanisms of job-search intensity and clarity should be explored in the future.

## Data Availability Statement

The raw data supporting the conclusions of this article will be made available by the authors, without undue reservation.

## Ethics Statement

This study was reviewed and approved by University of Electronic Science and Technology, China. Written informed consent for participation was not required for this study in accordance with the national legislation and the institutional requirements. The study was carried out in accordance with the Declaration of Helsinki.

## Author Contributions

YY wrote the manuscript and analyzed the data under the guidance of XY. XY contributed to the study design and critical revisions. All authors contributed to the article and approved the submitted version.

## Conflict of Interest

The authors declare that the research was conducted in the absence of any commercial or financial relationships that could be construed as a potential conflict of interest.

## Publisher’s Note

All claims expressed in this article are solely those of the authors and do not necessarily represent those of their affiliated organizations, or those of the publisher, the editors and the reviewers. Any product that may be evaluated in this article, or claim that may be made by its manufacturer, is not guaranteed or endorsed by the publisher.
